# Altered Body Composition and Cytokine Production in Patients with Elevated HOMA-IR after SARS-CoV-2 Infection: A 12-Month Longitudinal Study

**DOI:** 10.3390/biomedicines12071581

**Published:** 2024-07-17

**Authors:** Rona Kartika, Imam Subekti, Farid Kurniawan, Syahidatul Wafa, Tika Pradnjaparamita, Dicky L. Tahapary, Heri Wibowo

**Affiliations:** 1 Doctoral Program in Biomedical Sciences, Faculty of Medicine, Universitas Indonesia, Jl. Salemba Raya No.6, Jakarta 10430, Indonesia; rona.kartika11@ui.ac.id; 2 Metabolic Disorder, Cardiovascular, and Aging Research Center, Indonesia Medical Education & Research Institute (IMERI), Faculty of Medicine, Universitas Indonesia, Jl. Salemba Raya No.6, Jakarta 10430, Indonesia; farid.kurniawan01@ui.ac.id (F.K.); syahidatul.wafa@ui.ac.id (S.W.); tika.pradnjaparamita@yahoo.com (T.P.); dicky.tahapary@ui.ac.id (D.L.T.); 3 Division of Endocrinology, Metabolism, and Diabetes, Department of Internal Medicine, Cipto Mangunkusumo Hospital, Faculty of Medicine, Universitas Indonesia, Jl. P. Diponegoro No. 71, Jakarta 10430, Indonesia; imam.subekti@ui.ac.id; 4 Department of Parasitology, Faculty of Medicine, Universitas Indonesia, Jl. Salemba Raya No.6, Jakarta 10430, Indonesia

**Keywords:** post-COVID-19 condition, body composition, HOMA-IR, diabetes, cytokines, inflammation

## Abstract

Altered body composition and cytokine production due to SARS-CoV-2 antigens may affect homeostasis model assessment for insulin resistance (HOMA-IR) after SARS-CoV-2 infection. To elucidate this phenomenon, we conducted a longitudinal study involving 47 COVID-19 patients, who were followed up for 12 months. During recruitment, body composition and glucose indices were measured, and heparin blood samples were collected for measuring cytokine production. HOMA-IR was considered an elevated or non-elevated group based on the ratio between HOMA-IR at 12 months and 1 month of convalescence. Those with elevated HOMA-IR had a significantly higher body mass index, body fat percentage, and visceral fat rating and had a lower lean mass and lean/fat mass ratio than their counterparts. During the convalescent period, the elevated HOMA-IR group had lower TNFα, IFNγ, IL-2, IL-10, and granzyme B expression levels but had higher TNFα/IL-10, IFNγ/IL-10, IL-2/IL-10, and granzyme B/IL-10 ratios than the other group. The reduced cytokine production and pro-/anti-inflammatory imbalance in patients with elevated HOMA-IR may suggest immune cell dysfunction toward SARS-CoV-2. Patients with elevated HOMA-IR after SARS-CoV-2 infection may experience an increase in BMI and body fat percentage, leading to increased immune dysfunction and chronic inflammatory condition. A nutritional approach and promotion of physical activity may help reduce HOMA-IR and ameliorate glucose indices in these patients.

## 1. Introduction

The coronavirus disease 2019 (COVID-19) pandemic caused by severe acute respiratory syndrome coronavirus 2 (SARS-CoV-2) is over [1]. However, the pandemic has left us with another health burden—diabetes mellitus (DM) [2]. Growing evidence shows that the prevalence of DM has increased among individuals with a history of COVID-19 infection [3]. Recent meta-analyses and retrospective studies conducted in Europe and the United States showed a 40–67% increase in the risk of diabetes after COVID-19 infection [4,5,6], suggesting a bidirectional relationship between COVID-19 and diabetes [7]. Furthermore, COVID-19 reinfection also contributed to the increased risk of mortality with a hazard ratio (HR) of 2.17, 95% confidence interval (CI) = 1.93–2.45, and health sequelae including diabetes with HR = 1.70, 95%CI = 1.41–2.05 [8]. Not only DM, but the prevalence of insulin resistance, a pivotal risk factor for diabetes, also increased after COVID-19 infection [9,10].

SARS-CoV-2 infection affects not only the short-term but also the long-term immune system [11]. Studies showed that after SARS-CoV-2 infection, patients still experienced abnormal immune response and inflammation; thus, SARS-CoV-2 infection is correlated with the risk of inflammation-related diseases, such as autoimmune diseases and long COVID [11,12]. In long COVID, persistently elevated levels of cytokines—tumor necrosis factor (TNF)-α, interferon (IFN)-γ, interleukin (IL)-6, and IL-1β—were generated by an abnormal immune response, and these cytokines have been suggested to contribute to the pathogenic mechanism of long COVID [13,14].

Insulin resistance is associated with high serum levels of proinflammatory cytokines [15,16,17]. According to Al-Hakeim et al. [18], a high inflammatory response or a cytokine storm during acute SARS-CoV-2 infection is related to the development of long COVID-associated insulin resistance. This finding is consistent with that of Montefusco et al. [19] who reported that 18.5% of COVID-19 patients possibly had hyperglycemia and new-onset DM that was either transient or persistent. Another longitudinal study found that >50% of patients with new-onset diabetes due to COVID-19 still had DM 1 year after infection [20]. The persistence of insulin resistance or DM due to COVID-19 has not yet been fully understood. Similar to the pathogenic mechanism of long COVID, a persistent immune dysregulation after COVID-19 infection might have led to insulin resistance. Moreover, during the COVID-19 pandemic, which involved a series of lockdowns, the prevalence of obesity increased from 11% to 25.3% in men and from 15% to 42.4% in women [21]. Obesity, which is a major cause of insulin resistance, can also induce abnormal peripheral T cell function through genetic alteration [22]. Yazdanpanah et al. [23] demonstrated that SARS-CoV-2 infection significantly impacts adipogenesis and lipolysis. Their study reported a 2% increase in total body fat percentage in COVID-19 patients during the 1-month convalescence period. Therefore, the association between long-term immune response after COVID-19 infection and the risk of COVID-19-associated insulin resistance must be elucidated.

We speculate that post-COVID-19 immune system dysregulation accompanied by altered body composition during the COVID-19 pandemic has led to COVID-19-associated insulin resistance. In this study, we explore whether altered body composition and the immune response to SARS-CoV-2 challenge during the 12-month convalescence phase impact the risk of increased homeostatic model assessment for insulin resistance (HOMA-IR). To elaborate on these connections, in this study, firstly, we analyzed the metabolic risk factors for increased HOMA-IR, such as body fat and lean mass. Then, we analyzed the ability of immune cells to secrete cytokines and compared the results for patients with and without elevated HOMA-IR after 12 months of convalescence.

This study is the first to evaluate both body composition and the immune response to SARS-CoV-2 during the 12-month COVID-19 convalescence phase and elucidate the association between the changes in these parameters and the risk of increased HOMA-IR. The findings will hopefully elucidate how long-term immune system dysregulation after COVID-19 infection induces insulin resistance.

## 2. Materials and Methods

### 2.1. Study Design

This nested case–control study included participants from the COVID-19, Aging, and Cardiometabolic Risk Factors (CARAMEL) study with registration number NCT04802044 [23]. The CARAMEL study is a cohort study that investigated the impact of COVID-19 infection on aging and cardiometabolic risk factors. This study was approved by the ethics committee of the Faculty of Medicine, University of Indonesia-Cipto Mangunkusumo Hospital, Jakarta, Indonesia with reference number KET-1112/UN2.F1/ETIK/PPM.00.02/2020. All patients agreed to participate and signed the informed consent prior to sample collection.

### 2.2. Participants

The participants were patients who were admitted for COVID-19 infection at the Cipto Mangunkusumo Hospital, Jakarta, Indonesia, between 1 January 2021 and 31 March 2021. COVID-19 infection was confirmed using reverse transcription polymerase chain reaction wherein a cycle threshold of >24 indicates a positive result. The inclusion criteria were as follows: patients who contracted COVID-19 infection for the first time, did not receive any COVID-19 vaccine, and had completed all follow-up sessions. The exclusion criteria were as follows: aged <18 years, pregnant, and a history of malignant, autoimmune, and blood clotting disorders. This study consisted of 5 time points—1 time point at baseline (acute phase) and 4 time points at 1, 3, 9, and 12 months of the convalescence phase.

Baseline (acute phase) data, including symptoms, comorbidities, vaccination status, vital signs, and body composition, were collected upon hospital admission of eligible participants. Then, the participants were asked to fast overnight. Blood samples were collected 1 day after hospital admission. Sociodemographic characteristics, COVID-19 severity, and clinical and laboratory data were extracted from electronic medical reports.

In this longitudinal study, body composition and glycemic indices—fasting blood glucose (FBG), HbA1c, insulin, and C-peptide levels—were evaluated at four time points: 1, 3, 9, and 12 months after hospital admission. Cytokine production after SARS-CoV-2 challenge was measured from the peripheral blood mononuclear cell (PBMC) samples collected at the acute phase and at 3, 9, and 12 months. COVID-19 sequelae symptoms and vaccination status were determined at each follow-up session.

The participants were assigned to either the elevated or non-elevated HOMA-IR group based on the ratio of HOMA-IR at 12 months to that at 1 month (HOMA-IR 12/HOMA-IR 1). The median HOMA-IR 12/HOMA-IR 1 was 1.22, which served as the cut-off, that is, HOMA-IR 12/HOMA-IR 1 values of >1.22 and ≤1.22 indicated elevated and non-elevated HOMA-IR, respectively. The participants with and without elevated HOMA-IR comprised the case and control groups, respectively.

### 2.3. Body Composition Measurement

Body composition, which consists of body weight, BMI, body fat percentage, lean mass, and visceral fat rating, was assessed in a standing position through bioelectric impedance analysis by using a segmental body composition monitor (Tanita Model BC-601, Tanta Corp., Tokyo, Japan). The measurement was performed in the acute phase and at 1, 3, 9, and 12 months after COVID-19 infection.

### 2.4. FBG and HbA1c Measurements

Fasting venous blood samples were collected from all participants who fasted for 8–12 h prior to blood collection. Fasting blood glucose was measured using an Accu Check Performa for Blood Glucose (Roche Diabetes Care, Mannheim, Germany), and HbA1c was measured using a D-10 HbA1c analyzer (Bio-Rad, Hercules, CA, USA). Fasting plasma and serum samples were collected at five-time points and then frozen at −80 °C for further measurements.

### 2.5. Insulin and C-Peptide Measurements

Insulin and C-peptide were measured from fasting plasma samples using a DRG Insulin ELISA kit and a DRG C-peptide ELISA kit (DRG, Marburg, Germany), respectively. In brief, each sample was added into pre-coated microtiter wells and then incubated with an enzyme conjugate and antiserum (for C-peptide only). After 1 h of incubation, the unbound conjugates were washed off, and then streptavidin–peroxidase enzyme complex was added into each microtiter well. After the substrate solution was added, absorbance was measured using a VARIOSKAN LUX Multimode Microplate Reader (Thermo Fisher Scientific, Waltham, MA, USA) at 450 nm.

### 2.6. HOMA-IR and HOMA-B Calculation

HOMA1-IR was calculated using the formula used by Matthew et al. [20]: HOMA-IR = plasma glucose (mg/dL) × insulin (µIU/mL)/405. HOMA-B was also calculated to estimate beta cell function by using the following formula: 20 × fasting insulin (µIU/mL)/FBG (mg/dL). The normal range for HOMA-B is 70–150% [21]. HOMA-B < 70 indicates beta cell dysfunction, whereas HOMA-B > 150 indicates hyperinsulinemia.

### 2.7. Cell Stimulation

PBMCs were isolated through gradient centrifugation with Ficoll–Paque Plus (Cytiva, Uppsala, Sweden). After being isolated, the cells were resuspended in a cryomedium and stored in liquid nitrogen. At the time of stimulation, the cell suspension was thawed in a 37 °C water bath until only a small ice cube remained. The cell suspension was subsequently transferred to a warm complete medium containing RPMI 1640 (Gibco, Grand Island, NY, USA), 10% heat-inactivated fetal bovine serum (FBS, Gibco, Grand Island NY, USA), and 1% penicillin–streptomycin (Gibco, Grand Island, NY, USA). The cells were centrifuged at 400× *g* for 10 min; subsequently, they were resuspended using 1 mL complete medium and then counted. Approximately 1 × 10^6^ cells in 100 µL culture medium were seeded in a 96-well culture plate and stimulated using 2 µg/mL cocktail overlapping peptide (OLP) SARS-CoV-2 Protein-S and N (Milteny Biotec, Gladbach, Germany) for 24 h.

For quality control, each sample was seeded and cultured for 24 h with two different stimuli: 0.2 µL cell stimulation cocktail (Invitrogen, Carlsbed, CA, USA) as the positive control and complete medium as the negative control. The cells were harvested, and the supernatant was used for cytokine measurement. The capability of the thawed cells to produce cytokines was assessed using the positive control. The samples that failed to produce cytokines following stimulation were discarded. The negative control was used to assess non-specific stimulation that induces cytokine production. The cytokine data represent the differences between the values representing the response to the OLP SARS-CoV-2 antigen and the negative control.

### 2.8. Cytokine Measurement

The cytokines TNFα, IFNγ, IL-2, IL-10, and granzyme B were measured from the supernatant of the PBMC culture. These cytokines were measured using a bead-based multiplex assay with a Human Luminex Discovery Assay kit (R&D System, Minneapolis MN, USA). According to the manufacturer’s protocol, 25 µL samples or standards were added into a 96-well plate and mixed with a magnetic bead cocktail. The well plate was incubated with agitation for 2 h at room temperature (RT). After incubation, the well plate was washed three times using 200 µL wash buffer. Next, 25 µL detection antibody was added and incubated with agitation for 1 h at RT. Then, 25 µL streptavidin was added and incubated for 30 min. The well plate was washed and added with 200 µL wash buffer and prepared for analysis using Luminex 200 (Luminex, Minneapolis, MN, USA).

### 2.9. Statistical Analysis

Data were analyzed using IBM-Statistical Package for the Social Sciences version 24.0 for Windows (IBM SPSS, IBM Corp., Armonk, NY, USA). Data distribution was analyzed using the Kolmogorov–Smirnov test. For normally distributed data, continuous variables are presented as mean ± standard deviation and analyzed using the independent T-test. For data with non-normal distributions, data are presented as median (minimum–maximum) and analyzed using the Mann–Whitney test. Categorical variables are presented as proportions and were compared using the chi-square test. A repeated ANOVA followed by a Bonferroni comparison or a Friedman test followed by a Wilcoxon signed-rank test was used to analyze the differences among the time points. GraphPad Prism 8 (GraphPad Software, Inc., San Diego, CA, USA) was used to generate graphs for data visualization.

## 3. Results

### 3.1. Sociodemographic Characteristics

The participants were considered as having elevated HOMA-IR if their HOMA-IR 12/HOMA-IR 1 ratio was >1.22 or if there was a 22% increase in HOMA-IR during the 12 months of follow-up. The sociodemographic characteristics of the 47 participants, of whom 23 had non-elevated HOMA-IR and 24 had elevated HOMA-IR, were assessed. Table 1 showed no differences between the two groups in terms of gender, age, COVID-19 severities, comorbidities, and COVID-19 vaccination status.

### 3.2. Body Composition of the Participants during the 12-Month Convalescent Period

Expectedly, the HOMA-IR index was influenced by body composition. The patients with elevated HOMA-IR had a significantly higher BMI, body fat percentage, and visceral fat rating, as well as a lower lean mass and lean/fat mass ratio than those without elevated HOMA-IR. These differences became more noticeable at 3 months of follow-up. For those with elevated HOMA-IR, their BMI significantly increased by 1 kg/m^2^ (*p* < 0.001) and their body fat significantly increased by nearly 2% (*p* = 0.044) during the 12-month follow-up (Table 2). By contrast, those with non-elevated HOMA-IR showed a 3% decrease in body fat and 3% increase in lean mass. Moreover, during the 12-month follow-up, a decrease in body fat percentage and an increase in lean mass and lean/fat mass ratio were observed only in the non-elevated HOMA-IR group with *p* = 0.012, 0.070, and 0.002, respectively.

### 3.3. Glucose Indices during the 12-Month Convalescent Period

Regarding the glucose indices, besides HOMA-IR, the levels of HbA1c, fasting insulin, and fasting C-peptide, as well as HOMA-B, were also significantly increased (*p* < 0.001) in the elevated HOMA-IR group during the 12-month follow-up. Meanwhile, the non-elevated HOMA-IR group exhibited improved glycemic control marked by a reduced HbA1c level and HOMA-IR index during the 12-month follow-up (Table 3). Although beta cell function was regained after 12 months, beta cell dysfunction was observed in the elevated HOMA-IR group during the acute phase, with a median HOMA-B index of 52.55%.

### 3.4. Cytokine Production during the 12-Month Convalescent Period

**Figure 1:**
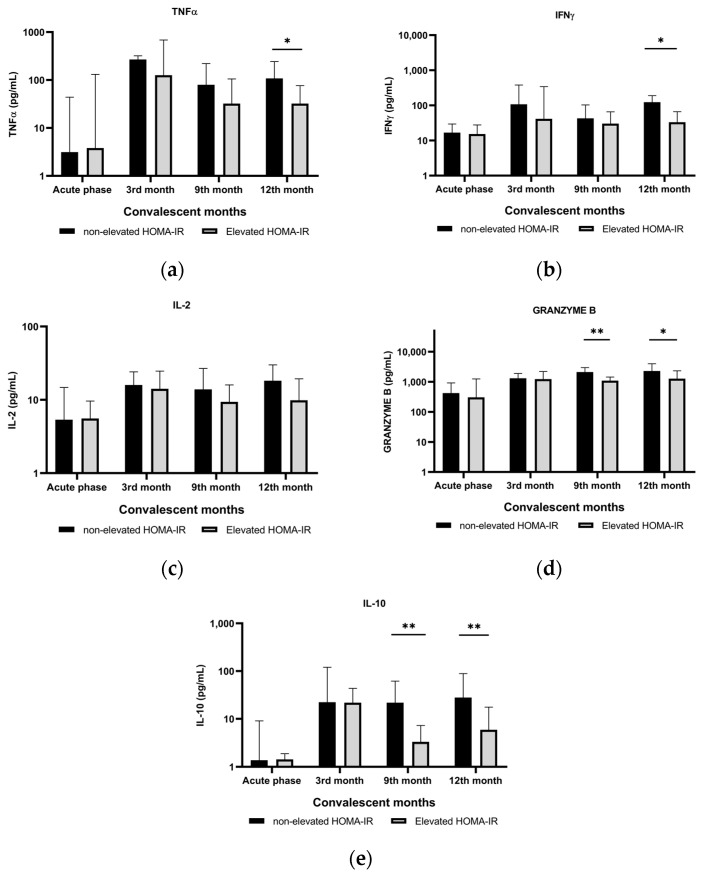
Secretion levels of (**a**) TNFα, (**b**) IFNγ, (**c**) IL-2, (**d**) granzyme B, and (**e**) IL-10 detected from the SARS-CoV-2-stimulated supernatant obtained from convalescent COVID-19 patients. Black bars represent COVID-19 convalescent patients with non-elevated HOMA-IR, and gray bars represent those with elevated HOMA-IR. Acute phase indicates that the samples were obtained during acute COVID-19 infection. * *p* value < 0.05 and ** *p* value < 0.01. TNF-α, tumor necrosis factor α; IFNγ, interferon γ; IL-2, interleukin-2; IL-10, interleukin-10. All cytokine levels are presented as median (interquartile range) and were compared using the Mann–Whitney test.

Cytokine levels were measured from the supernatant of PBMCs stimulated with OLP SARS-CoV-2 Protein-S and N for 24 h. Figure 1 shows no differences in both pro- and anti-inflammatory cytokine production were observed between the elevated and non-elevated HOMA-IR groups during acute COVID-19 infection (acute phase). However, during the convalescent period (3–12 months after COVID-19 infection), the production of all cytokines was lower in the elevated HOMA-IR group than in the non-elevated HOMA-IR group. The differences were more evident for granzyme B and IL-10 at 9 and 12 months. Meanwhile, differences in the TNFα and IFNγ levels were observed only at 12 months. We also analyzed the fluctuation in cytokine production during the convalescent phase and found no changes in the TNFα, IFNγ, IL-2, and IL-10 levels in the non-elevated HOMA IR group. Compared with the other cytokines, granzyme B showed a significant increase at *p* = 0.005. These lines of evidence indicate that throughout the 12-month convalescent period, the cells were producing the same level of protection after SARS-CoV-2 challenge. However, during the same period, in the elevated HOMA-IR group, the TNFα and IL-10 production levels gradually decreased at *p* = 0.023 and 0.018, respectively, and this phenomenon may correlate with immune cell dysfunction toward SARS-CoV-2 challenge (Supplementary Material S4).

### 3.5. Pro-/Anti-Inflammation Ratio during the 12-Month Convalescent Period

In eliminating pathogens, cytokines are orchestrated to achieve the optimal level that can induce inflammation but at the same time prevent tissue destruction due to an excessive pro-inflammatory response. IL-10 is a very potent anti-inflammatory cytokine that not only controls inflammatory response but also induces tissue regeneration. We explored the role of IL-10 by comparing IL-10 to pro-inflammatory cytokines, such as TNFα, IFNγ, IL-2, and granzyme B.

**Figure 2:**
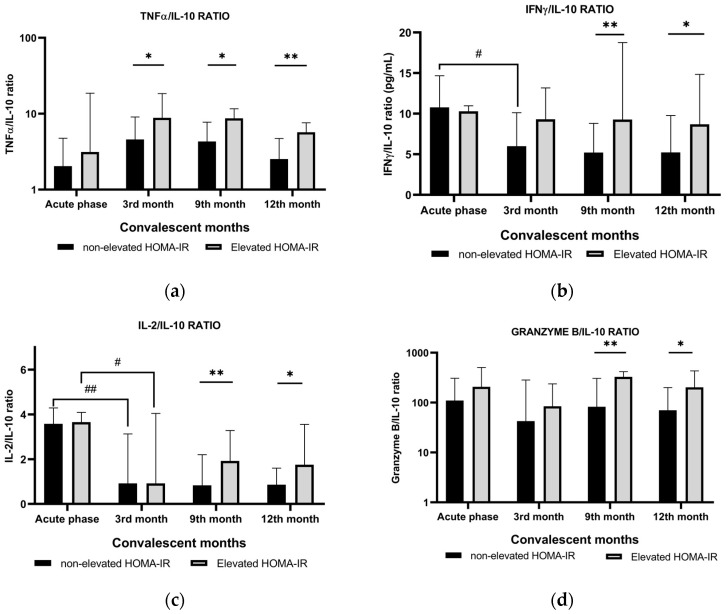
(**a**) TNFα/IL-10, (**b**) IFNγ/IL-10, (**c**) IL-2/IL-10, and (**d**) granzyme B/IL-10 ratios detected in the SARS-CoV-2-stimulated supernatant of the samples obtained from the convalescent COVID-19 patients. Black bars represent convalescent COVID-19 patients with non-elevated HOMA-IR, and gray bars represent those with elevated HOMA-IR. Acute phase indicates that the samples were obtained during acute COVID-19 infection. * *p* value < 0.05 and ** *p* value < 0.01. # *p* value < 0.05 and ## *p* value < 0.01 compared with the acute phase. TNF-α, tumor necrosis factor α; IFNγ, interferon γ; IL-2, interleukin-2; IL-10, interleukin-10. All cytokine levels are presented as median (interquartile range) and were compared using the Mann–Whitney test. Differences in IFNγ/IL-10 and L-2/IL-10 ratios between the acute phase and 3rd convalescent month were analyzed using the Wilcoxon signed-rank test.

Figure 2 shows that during the acute phase, as a response to infection, the IFNγ/IL-10 and IL-2/IL-10 ratios were significantly higher (*p* = 0.049 and 0.004, respectively) compared with those at 3 months of convalescence, indicating Th1 dominance. At 3 months through 12 months of convalescence, the TNFα/IL-10, IFNγ/IL-10, IL-2/IL-10, and granzyme B/IL-10 ratios were lower in the non-elevated HOMA-IR group than in the elevated HOMA-IR group. These results demonstrate that in the non-elevated HOMA-IR group, anti-inflammatory cytokines could alleviate the inflammatory process during SARS-CoV-2 challenge or reinfection, minimizing tissue destruction despite the high production of pro-inflammatory cytokines. During the convalescent period, the elevated HOMA-IR group had higher TNFα/IL-10, IFNγ/IL-10, IL-2/IL-10, and granzyme B/IL-10 ratios than the non-elevated HOMA-IR group, suggesting that low IL-10 production could not control the production of pro-inflammatory cytokines, and this phenomenon may induce chronic inflammation.

Interestingly, 52% of the patients in the non-elevated HOMA-IR group had diabetes. We found that in this group, patients with diabetes had an immune response similar to that of patients without diabetes in the non-elevated HOMA-IR group. In the non-elevated group, the patients with diabetes had high pro-inflammatory cytokine levels accompanied by high anti-inflammatory cytokine levels, an indication of a low inflammatory response (Supplementary Material S5).

## 4. Discussion

After the remission of acute COVID-19 infection, some patients reported an increase in blood glucose, or individuals with type 2 DM reported an uncontrolled glycemic level. Recent evidence showed that the risk of newly diagnosed DM increased after COVID-19 infection [3], and one-third of patients who had recovered from COVID-19 exhibited elevated glucose indices marked by an increase in HOMA-IR, FBG, and insulin levels [18]. In this study, the mean change in HOMA-IR during the 12-month convalescent period was 22%. Individuals who had at least a 22% increase in HOMA-IR index after COVID-19 infection had a higher body fat percentage and higher glycemic indices than those with less than a 22% increase in HOMA-IR index (non-elevated HOMA-IR). Improvement in body composition, which is marked by reduced body fat and increased lean mass, seems to have contributed to the restoration of the HbA1c and HOMA-IR indices. Elevated HOMA-IR also influenced cytokine production after SARS-CoV-2 challenge. We found that individuals with elevated HOMA-IR after COVID-19 infection had low levels of pro- and anti-inflammatory cytokines, which are needed for protection. Furthermore, low IL-10 production promotes pro-/anti-inflammatory cytokine imbalance, which may induce chronic inflammation.

### 4.1. Glycometabolic Control Influences HOMA-IR Elevation

COVID-19 has been associated with altered glucose metabolism and increased HOMA-IR [10]. One mechanism that induces an increase in HOMA-IR involves BMI and body fat [24,25]. One year after acute COVID-19 remission, patients who had severe COVID-19 experienced an increase in body fat and BMI with worsened physical activity [26]. Yazdanpanah et al. [27] also reported an increase in total body fat percentage by nearly 2% after 1 month of COVID-19 remission, and this phenomenon was followed by an increase in HOMA-IR index. Adatsi et al. [28] also reported that in Ghana, after 7 months, patients who had recovered from COVID-19 showed increased insulin resistance and decreased beta cell function. Consistent with these findings, our results showed that recovered COVID-19 patients with elevated HOMA-IR had a 1 kg/m^2^ increase in BMI and a 2% increase in total body fat during the 12-month follow-up. Meanwhile, those who recovered from COVID-19 but without elevated HOMA-IR had a 3% decrease in body fat and a significant increase in lean/fat mass ratio even though their BMI values did not change. Increases in body fat and BMI after COVID-19 are strongly associated with lower physical activity, increased calorie intake [29], and sedentary behavior during the COVID-19 pandemic [30]. However, we did not obtain data on physical activity and calorie intake, but we believe that the work-from-home setup, the nationwide lockdowns [31], and changes in food consumption patterns during COVID-19 in Indonesia, Ref. [32] have contributed to the increase in BMI and body fat percentage, resulting in increased HOMA-IR.

Apart from HOMA-IR, the long-term disruption in the glycometabolic control in the elevated HOMA-IR group was also examined. Fasting insulin, fasting C-peptide, and HOMA-IR gradually increased at 3–12 months of the convalescent period, but the FBG level remained the same. Similar to our findings, Chen et al.’s [10] results for COVID-19 patients showed that compared with the baseline, the levels of fasting C-peptide, HOMA-B, HOMA-IR, and triglyceride glucose index significantly increased, whereas FBG decreased at 6 months of follow-up. This diabetogenic effect of COVID-19 may have been due to beta cell dysfunction, disturbance of adiponectin/leptin level that can dysregulate the glucose level, and systemic inflammation [33].

### 4.2. Immune Dysfunction Contributes to HOMA-IR Elevation

Regarding the immune system, we determined how the immune cells of patients with and without elevated HOMA-IR react to SARS-CoV-2. This study showed that the immune response toward the SARS-CoV-2 antigen in patients with elevated HOMA-IR was dampened, and this finding was evident until 12 months of follow-up. Our finding reveals that even in a pre-diabetic condition, a low immune response toward infection under elevated HOMA-IR was similar to that observed in diabetic patients. Immune dysfunction marked by low cytokine secretion after stimulation in diabetics was previously reported [34]. Similarly, our previous study showed that after phytohemagglutinin stimulation, the PBMCs of patients with diabetes expressed lower IFNγ and IL-10 levels; however, they had higher monocyte responsiveness, which is measured based on TNFα/IFNγ and IL-6/IFNγ ratios [34].

Granzyme B is a serine protease that contributes to cell killing. In insulin resistance and type 2 diabetes, circulatory granzyme B was increased, and it is a component of an inflammatory marker [35]; Yoon Kim et al. [36] showed that reduced cytotoxic expression and a low amount of apoptosis inducer were observed in natural killer cells in diabetic mice. Similar to the findings of Yoon Kim [36], our results showed that the elevated HOMA-IR group had a persistently low granzyme B production after antigen stimulation, suggesting a low cytotoxic activity.

IL-10 is a potent anti-inflammatory cytokine that can control inflammation and induce tissue healing, and its production is reduced in chronic inflammation, such as atherosclerosis [37] and multiple sclerosis [38]. We found that the elevated HOMA-IR group had persistently low IL-10 levels during the 12-month follow-up accompanied by a high inflammatory response characterized by high TNFα/IL-10, IFNγ/IL-10, IL-2/IL-10, and granzyme B/IL-10 ratios. Similar to what has been observed in patients with diabetes, high glucose levels can impair the IL-10 signaling protein—signal transducer and activator of transcription 3 (STAT3)—and lead to IL-10 hyporesponsiveness [39]. This condition also contributes to chronic inflammation [39].

In this study, even for DM patients, the maintenance of body composition promotes normal HOMA-IR and HbA levels, which in turn regulate body fat and increase lean mass. In DM patients without elevated HOMA-IR, memory cells maintain the production of a good amount of pro- and anti-inflammatory cytokines but at the same time prevent an excessive pro-inflammatory response and chronic inflammation.

The strengths of this study include the measurement of the long-term impact of COVID-19 on body composition, glucose indices, and immune response toward SARS-CoV-2 antigens during the acute phase and at four time points of follow-up during the COVID-19 convalescent period. Moreover, patients with diabetes were included in this study; hence, we could estimate the risk of body composition and the changes in immune response during the course of the study. However, this study failed to include healthy control subjects who had no SARS-CoV-2 infection, had no vaccination during the height of the pandemic, and had no vaccination during the observation period.

## 5. Conclusions

Patients with elevated HOMA-IR, regardless of their diabetes status, had a significantly higher BMI, body fat percentage, and visceral fat rating as well as lower lean mass and lean/fat mass ratio than patients without elevated HOMA-IR. Regarding glucose indices, HbA1c, fasting insulin, fasting C-peptide, and HOMA-B levels were also significantly higher during the 12-month follow-up. During the SARS-CoV-2 challenge, cytokine secretion markedly decreased, whereas inflammatory response increased. These results indicate that those with elevated HOMA-IR after SARS-CoV-2 infection had reduced cytokine secretion, which influenced their long-term immunity; moreover, they had increased inflammatory responses that could worsen their insulin sensitivity. A nutritional approach and promotion of physical activity may help reduce HOMA-IR and may ameliorate glucose indices in these patients.

## Figures and Tables

**Table 1:** Sociodemographic characteristics of the participants.

Parameters	Non-Elevated HOMA-IR (n = 23)	Elevated HOMA-IR (n = 24)	*p* Value
Gender			0.765
Male (n, %)	15 (65.2%)	16 (66.6%)	
Female (n, %)	8 (34.8%)	8 (33.3%)	
Age (years)	47.78 ± 10.69	47.46 ± 12.39	0.929
COVID-19 Severity			
Asymptomatic (n, %)	2 (8.7%)	3 (12.5%)	0.521
Mild (n, %)	4 (17.4%)	6 (25%)	0.391
Moderate (n, %)	12 (52.2%)	8 (33.3%)	0.156
Severe (n, %)	4 (17.4%)	6 (25%)	0.391
Critical (n, %)	1 (4.3%)	1 (4.2%)	0.745
Comorbidities			
Diabetes (n, %)	12 (52.1%)	10 (41.6%)	0.708
Hypertension (n, %)	9 (39.1%)	9 (37.5%)	0.573
Kidney Failure (n, %)	1 (4.3%)	2 (8.3%)	0.516
Heart Failure (n, %)	3 (13%)	2 (8.3%)	0.479
Dyslipidemia (n, %)	7 (30.4%)	5 (20.8%)	0.410
Stroke (n, %)	2 (8.6%)	0	0.234
COPD (n, %)	1 (4.3%)	1 (4.1%)	0.745
Asthma (n, %)	2 (8.6%)	4 (16.6%)	0.354
COVID-19 Vaccination Status			
3rd month	7 (30.4%)	6 (25%)	0.677
9th month	20 (87%)	22 (91.3%)	0.495
12th month	22 (91.7%)	23 (95.8%)	0.792

COVID-19, coronavirus disease 2019; n, sample size; COPD, chronic obstructive pulmonary disease. Age is presented as mean ± standard deviation and analyzed using independent T-test. Gender, severity of COVID-19 infection, comorbidities, and COVID-19 vaccination status are presented as number (percentages) and analyzed using the chi-square test. The frequency of COVID-19 vaccination at each time point is detailed in Supplementary Material S1.

**Table 2:** Body composition of the participants during the 12-month convalescent period.

Parameters	Non-Elevated HOMA-IR (n = 23)	Elevated HOMA-IR (n = 24)	*p* Value ^a^
BMI (kg/m^2^)			
acute phase	25.98 ± 3.69	30.38 ± 5.25	0.017 *
12th month	26.3 ± 3.45	31.37 ± 5.13	0.005 **
*p* value ^b^	0.640	<0.001 ***	
Body fat (%)			
acute phase	30.27 ± 9.33	33.87 ± 8.24	0.168
12th month	27.73 ± 9.08	35.67 ± 10.04	0.017 *
*p* value ^b^	0.012 *	0.044 *	
Lean mass (%)			
acute phase	65.39 ± 8.64	61.64 ± 9.60	0.168
12th month	68.38 ± 8.75	58.81 ± 9.67	0.019 *
*p* value ^b^	0.070	0.065	
Visceral fat rating			
acute phase	11.09 ± 4.85	15.12 ± 4.72	0.006 **
12th month	10.7 ± 4.92	14.45 ± 4.27	0.009 *
*p* value ^b^	0.343	0.076	
Lean/fat mass ratio			
acute phase	2.34 (0.91–5.73)	1.89 (0.8–3.5)	0.233
12th month	2.51 (1.16–8.83)	1.66 (0.77–3.4)	0.039 *
*p* value ^b^	0.002 **	0.165	

^a^ Statistical value for a cross-sectional study; ^b^ statistical value for a longitudinal study; * *p* value < 0.05; ** *p* value < 0.01; *** *p* value < 0.001. Acute phase indicates that the samples were obtained during acute COVID-19 infection. BMI, body mass index; BMI, body fat percentage, lean mass, and visceral fat rating are presented as mean ± standard deviation and were analyzed using the independent T-test. The lean/fat mass ratios are presented as median (minimum–maximum) and were compared using the Mann–Whitney test. For longitudinal analysis, the differences among the time points were analyzed using the repeated measures ANOVA for BMI, body fat percentage, lean mass, and visceral fat rating. The lean/fat mass ratio was determined using the Friedman test. The data of metabolic parameters and their significant values from 1st, 3rd, and 9th month of convalescence phase are described in Supplementary Material S2.

**Table 3:** Glucose indices of the participants during the 12-month convalescent period.

Parameters	Non-Elevated HOMA-IR (n = 23)	Elevated HOMA-IR (n = 24)	*p* Value ^a^
HbA1c (%)			
acute phase	6.2 (5.3–12.4)	6.45 (5.2–13.8)	0.974
12th month	5.7 (5.0–10.8)	6.6 (5.2–10.5)	0.039 *
*p* value ^b^	<0.001 ***	<0.001 ***	
FBG (mg/dL)			
acute phase	102 (73–509)	126 (73–256)	0.530
12th month	104 (81–210)	118.50 (80–248)	0.069
*p* value ^b^	0.055	0.080	
Fasting Insulin (µIU/mL)			
acute phase	9.97 (2.28–32.13)	10.26 (3.35–66.2)	0.371
12th month	7.90 (2.91–24.97)	14.70 (6.35–47.87)	<0.001 ***
*p* value ^b^	0.676	<0.001 ***	
Fasting C-Peptide (ng/mL)			
acute phase	2.00 (0.56–4.32)	1.31 (0.64–5.66)	0.217
12th month	2.01 (0.61–4.89)	2.32 (0.24–7.19)	0.187
*p* value ^b^	0.002 **	<0.001 ***	
HOMA-IR Index			
acute phase	3.24 (0.832–20.77)	3.41 (0.75–17.98)	0.468
12th month	2.13 (0.61–9.18)	5.11 (1.66–21.04)	<0.001 ***
*p* value ^b^	0.312	<0.001 ***	
HOMA-B (%)			
acute phase	82.1 (5.2–200.4)	52.55 (15.5–222.8)	0.268
12th month	92.7 (16.9–163.5)	91.3 (14.3–188.6)	0.328
*p* value ^b^	0.012 *	<0.001 ***	

^a^ Statistical value for a cross-sectional study; ^b^ statistical value for a longitudinal study; * *p* value < 0.05; ** *p* value < 0.01; *** *p* value < 0.001. Acute phase indicates that the samples were obtained during acute COVID-19 infection. HbA1c, glycated hemoglobin; FBG, fasting blood glucose; HOMA-IR, homeostatic model assessment for insulin resistance; HOMA-B, homeostatic model assessment for beta cell function. All data are presented as median (minimum–maximum) and were compared using the Mann–Whitney test. For longitudinal analysis, differences among the time points were analyzed using the Friedman test. The data of glycemic indices and their significant values from 1st, 3rd, and 9th month of convalescence phase are described in Supplementary Material S3.

## Data Availability

The authors confirm that the data supporting the findings of this study are available within the article and its Supplementary material. Raw data that support the findings of this study are available from the corresponding author, upon reasonable request.

## Supplementary Materials

The following supporting information can be downloaded at: https://www.mdpi.com/article/10.3390/biomedicines12071581/s1, Table S1. COVID-19 Vaccination Status of the participants, Table S2. Body composition of the participants during the 12-month convalescent period, Table S3. Glucose indices of the participants during the 12-month convalescent period. Table S4. The alteration of cytokines production from 3rd to 12th month of COVID-19 convalescence period. Figure S1. Comparison of the levels of (a) TNFα, (b) IFNγ, (c) IL-2, (d) granzyme B, and (e) IL-10; and (f) TNFα/IL-10, (g) IFNγ/IL-10, (h) IL-2/IL-10, and (i) granzyme B/IL-10 ratios detected in the SARS-CoV-2-stimulated supernatant of the samples obtained from the convalescent COVID-19 patients with non-elevated HOMA-IR between non-diabetic and diabetic patients.
