# Supplementary material for: Altered Body Composition and Cytokine Production in Patients with Elevated HOMA-IR after SARS-CoV-2 Infection: A 12-Month Longitudinal Study
Source: Biomedicines. 2024 Jul 17;12(7):1581. doi: 10.3390/biomedicines12071581 (PMC11274364; doi:10.3390/biomedicines12071581)
Supplement: Supplementary file 1 [file biomedicines-12-01581-s001.zip › biomedicines-3038927-supplementary.pdf]

Supplementary Material S1

Table S1. COVID-19 vaccination status of the participants.

Parameters	non-elevated HOMA-IR (n=23)	elevated HOMA-IR (n=24)	p value
**COVID-19 Vaccination Status**			
**3rd month**			**0.677**
Never	16 (69.6%)	18 (75%)	
Once	7 (30.4%)	6 (25%)	
**9th month**			0.495
never	3 (13%)	2 (8.3%)	
Once	1 (4.3%)	0	
Twice	11 (47.8%)	16 (66.7%)	
3 times	8 (34.8%)	6 (25%)	
**12th month**			0.792
never	2 (8.7%)	1 (4.2%)	
Twice	12 (52.2%)	14 (58.3%)	
3 times	9 (39.1%)	9 (37.5%)	

n, sample size; COVID-19 vaccination status is presented as number (percentages) and analyzed using the chi-square test.

Supplementary Material S2

Table S2. Body composition of the participants during the 12-month convalescent period.

Parameters	non-elevated HOMA-IR (n=23)	elevated HOMA-IR (n=24)	p value[a]
**BMI (kg/m^2)**			
acute phase	25.98 ± 3.69	30.38 ± 5.25	0.017*
1st month	26.35 ± 3.55	30.39 ± 5.33	0.043*
3rd month	26.26 ± 3.81	30.56 ± 5.04	0.026*
9th month	26.26 ± 3.54	31.02 ± 5.09	0.007**
12th month	26.3 ± 3.45	31.37 ± 5.13	0.005**
p value[b]	0.640	<0.001***	
**Body fat (%)**			
acute phase	30.27 ± 9.33	33.87 ± 8.24	0.168
1st month	28.07 ± 7.79	33.07 ± 10.35	0.069
3rd month	28.38 ± 8.69	35.44 ± 9.69	0.029*
9th month	27.74 ± 8.35	35.23 ± 9.98	0.020*
12th month	27.73 ± 9.08	35.67 ± 10.04	0.017*
p value[b]	0.012*	0.044*	
**Lean mass (%)**			
acute phase	65.39 ± 8.64	61.64 ± 9.60	0.168
1st month	68.5 ± 9.39	63.19 ± 9.95	0.067
3rd month	67.8 ± 8.42	59.98 ± 9.36	0.030*
9th month	68.36 ± 8.06	59.2 ± 9.62	0.022*
12th month	68.38 ± 8.75	58.81 ± 9.67	0.019*
p value[b]	0.070	0.065	
**Visceral fat rating**			
acute phase	11.09 ± 4.85	15.12 ± 4.72	0.006**
1st month	11.56 ± 4.51	13.87 ± 4.48	0.085

Parameters	non-elevated HOMA-IR (n=23)	elevated HOMA-IR (n=24)	p value[a]
3rd month	11.08 ± 4.99	14.33 ± 4.22	0.029*
9th month	11.00 ± 4.92	14.41 ± 4.38	0.016*
12th month	10.7 ± 4.92	14.45 ± 4.27	0.009*
p value[b]	0.343	0.076	
**Lean/fat mass ratio**			
acute phase	2.34 (0.91-5.73)	1.89 (0.8-3.5)	0.233
1st month	2.52 (1.04-6.82)	2.1 (0.77-4.04)	0.120
3rd month	2.58 (1.01-7.48	1.86 (0.76-3.52)	0.032*
9th month	2.46 (1.16-5.87)	1.64 (0.79-3.53)	0.028*
12th month	2.51 (1.16-8.83)	1.66 (0.77-3.4)	0.039*
p value[b]	0.002**	0.165	

[a] Statistical value for a cross-sectional study; [b] statistical value for a longitudinal study; *p value < 0.05; **p value < 0.01; ***p value < 0.001. Acute phase indicates that the samples were obtained during acute COVID-19 infection. DM, diabetes mellitus; BMI, body mass index; kg/m^2, kilogram per square meter. BMI, body fat percentage, lean mass, and visceral fat rating are presented as mean ± standard deviation and were analyzed using the independent T-test. The lean/fat mass ratios are presented as median (minimum–maximum) and were compared using the Mann–Whitney test. For longitudinal analysis, the differences among the time points were analyzed using the repeated measures ANOVA for BMI, body fat percentage, lean mass, and visceral fat rating. The lean/fat mass ratio was determined using the Friedman test.

Supplementary Material S3

Table S3. Glucose indices of the participants during the 12-month convalescent period.

Parameters	non-elevated HOMA-IR (n=23)	elevated HOMA-IR (n=24)	p value[a]
**HbA1c (%)**			
acute phase	6.2 (5.3-12.4)	6.45 (5.2-13.8)	0.974
1st month	6.1 (5.2-13.0)	6.35 (5.2-10.2)	0.456
3rd month	5.6 (4.8-10.1)	5.8 (4.7-9.1)	0.572
9th month	5.7 (5.0-10.1)	6.8 (5.1-10.5)	0.176
12th month	5.7 (5.0-10.8)	6.6 (5.2-10.5)	0.039*
p value[b]	<0.001***	<0.001***	
**FBG (mg/dL)**			
acute phase	102 (73-509)	126 (73-256)	0.530
1st month	105 (86-268)	106.5 (79-199)	0.924
3rd month	104 (82-236)	103 (80-196)	0.558
9th month	104 (84-216)	108 (87-340)	0.166
12th month	104 (81-210)	118.50 (80-248)	0.069
p value[b]	0.055	0.080	
**Fasting Insulin (μIU/mL)**			
acute phase	9.97 (2.28-32.13)	10.26 (3.35-66.2)	0.371
1st month	10.46 (2.35-31.81)	10.07 (3.79-27.52)	0.949
3rd month	8.81 (2.94-49.04)	14.09 (4.91-41.40)	0.032*
9th month	8.87 (2.90-46.51)	12.24 (5.63-36.81)	0.051
12th month	7.90 (2.91-24.97)	14.70 (6.35-47.87)	<0.001***
p value[b]	0.676	<0.001***	

Parameters	non-elevated HOMA-IR (n=23)	elevated HOMA-IR (n=24)	p value[a]
**Fasting C-Peptide (ng/mL)**			
acute phase	2.00(0.56-4.32)	1.31(0.64-5.66)	0.217
1st month	1.35(0.56-13.4)	1.48(0.78-2.70)	0.958
3rd month	1.90(0.923-9.53)	2.38(1.33-4.81)	0.077
9th month	1.55(0.48-10.97)	1.54(0.76-5.30)	0.848
12th month	2.01(0.61-4.89)	2.32(0.24-7.19)	0.187
p value[b]	0.002**	<0.001***	
**HOMA-IR Index**			
acute phase	3.24 (0.832-20.77)	3.41 (0.75-17.98)	0.468
1st month	2.77 (0.54-13.74)	2.82 (0.881-7.79)	0.983
3rd month	2..26 (0.661-15.13)	4.17 (1.31-17.89)	0.023*
9th month	2.43 (0.61-24.81)	3.49 (1.60-21.13)	0.041*
12th month	2.13 (0.61-9.18)	5.11 (1.66-21.04)	<0.001***
p value[b]	0.312	<0.001***	
**HOMA-B (%)**			
acute phase	82.1 (5.2-200.4)	52.55 (15.5-222.8)	0.268
1st month	68.6 (14.6-165.3)	69.45 (20.1-129)	0.733
3rd month	95.5 (20.9-205.6)	84.65 (35.1-165.5)	0.774
9th month	77.6 (24.8-236.5)	63.95 (12.5-212.0)	0.131
12th month	92.7 (16.9-163.5)	91.3 (14.3-188.6)	0.328
p value[b]	0.012*	<0.001***	

[a] Statistical value for a cross-sectional study; [b] statistical value for a longitudinal study; *p value < 0.05; **p value < 0.01; ***p value < 0.001. Acute phase indicates that the samples were obtained during acute COVID-19 infection. HbA1c, glycated hemoglobin; FBG, fasting blood glucose; HOMA-IR, homeostatic model assessment for insulin resistance; HOMA-B, homeostatic model assessment for beta cell function. All data are presented as median (minimum–maximum) and were compared using the Mann–Whitney test. For longitudinal analysis, differences among the time points were analyzed using the Friedman test.

Supplementary Material S4

Table S4. The alteration of cytokines production from 3rd to 12th month of COVID-19 convalescence period

Parameters	non-elevated HOMA-IR (n=23)	elevated HOMA-IR (n=24)	p value[a]
**TNFα (pg/mL)**			
3rd month	268.01 (1.38-2748.92)	125.97(1.28-2959.53)	0.915
9th month	79.82(1.48-341.05)	32.21(1.82-507.14)	0.110
12th month	108.00 (0.78-925.55)	32.23(1.66-397.81)	0.019*
p value[b]	0.337	0.023*	
**IFNγ (pg/mL)**			
3rd month	107.26(9.37-5901.22)	41.62(5.15-9573.2)	0.595
9th month	42.72(15.70-1352.23)	30.30(4.77-434.05)	0.209
12th month	123.60(5.61-2430.02)	33.28(10.89-1560.48)	0.049*
p value[b]	0.296	0.275	
**IL-2 (pg/mL)**			
3rd month	15.93(3.63-207.24)	14.14(5.03-211.03)	0.678
9th month	13.9(0.93-55.55)	9.4(2.77-55.55)	0.166

Parameters	non-elevated HOMA-IR (n=23)	elevated HOMA-IR (n=24)	p value[a]
12th month	18.23(3.67-105.05)	9.88(1.96-181.65)	0.109
p value[b]	0.401	0.197	
**Granzyme B (pg/mL)**			
3rd month	1321.12(144.73-5204.16)	1232.89(148.58-6887.33)	0.949
9th month	2114.14(116.17-4021.31)	1098.12(256.90-3046.16)	0.001**
12th month	2297.69(72.97-5376.50)	1279.41(96.64-5269.86)	0.047*
p value[b]	0.005*	0.513	
**IL-10 (pg/mL)**			
3rd month	22.37(1.11-553.28)	21.82(0.91-145.90)	0.516
9th month	21.76(1.02-133.11)	3.30(0.32-25.66)	0.002**
12th month	27.99(0.34-270.86)	5.92(0.34-36.01)	0.007**
p value[b]	0.154	0.018*	

[a] statistical value for cross sectional study; [b] statistical value for longitudinal study; **p* value < 0.05; ***p* value < 0.01. TNF-α, tumor necrosis factor α, IFNγ, interferon γ; Il-2, Interleukin 2, IL-10, Interleukin 10; pg/mL, picogram/millilitres. All cytokine levels are presented as median (minimum-maximum). For cross sectional study, all cytokines are analysis using Mann-Whitney test and for longitudinal study, the cytokines level fluctuation is analysis using Friedman test.

Supplementary Material S5

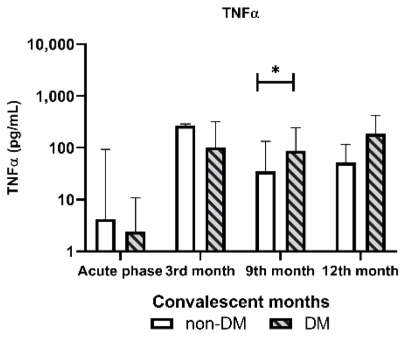

(a)

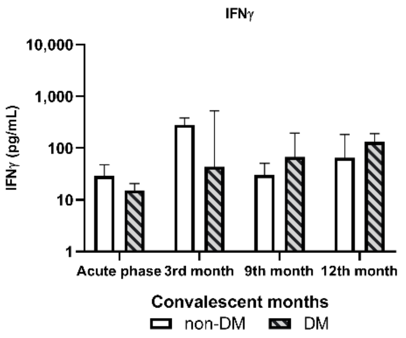

(b)

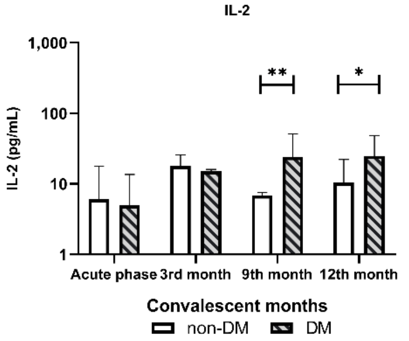

(c)

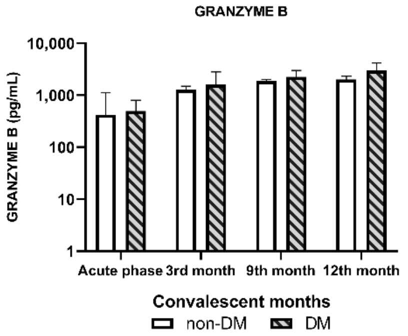

(d)

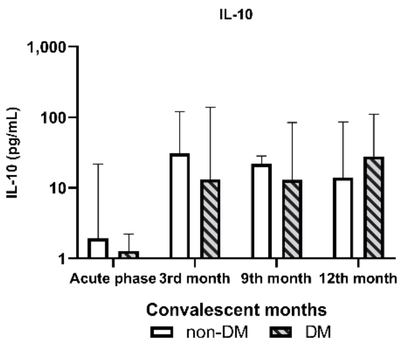

(e)

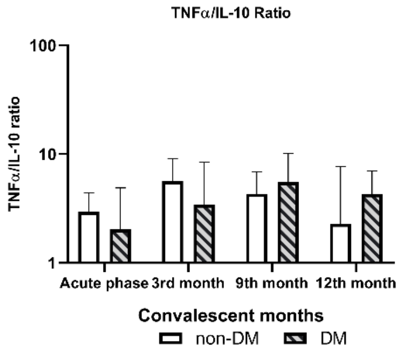

(f)

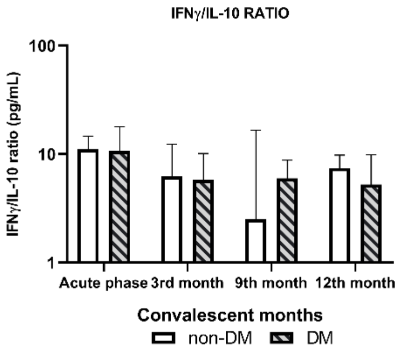

(g)

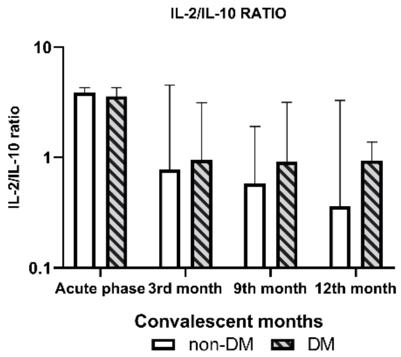

(h)

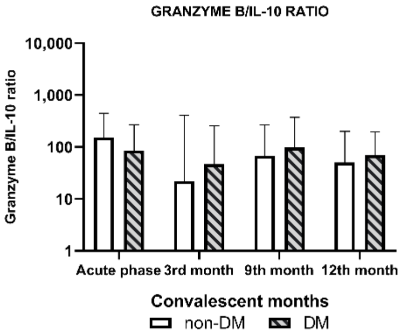

(i)

Figure S1. Comparison of the levels of (**a**) TNFα, (**b**) IFNγ, (**c**) IL-2, (**d**) granzyme B, and (**e**) IL-10; and (**f**) TNFα/IL-10, (**g**) IFNγ/IL-10, (**h**) IL-2/IL-10, and (**i**) granzyme B/IL-10 ratios detected in the SARS-CoV-2-stimulated supernatant of the samples obtained from the convalescent COVID-19 patients with non-elevated HOMA-IR between non-diabetic and diabetic patients. White bars represent convalescent COVID-19 patients with non-elevated HOMA-IR and non-diabetes and patterned bars represent those with diabetes. Acute phase indicates that the samples were obtained during acute COVID-19 infection. * p value < 0.05 and ** p value < 0.01. DM, diabetes mellitus, TNF-α, tumor necrosis factor α; IFNγ, interferon γ; IL-2, interleukin-2; IL-10, interleukin-10. All cytokine levels are presented as median (interquartile range) and were compared using the Mann–Whitney test.
